# Two-Stage Two-Locus Models in Genome-Wide Association

**DOI:** 10.1371/journal.pgen.0020157

**Published:** 2006-09-22

**Authors:** David M Evans, Jonathan Marchini, Andrew P Morris, Lon R Cardon

**Affiliations:** 1 Wellcome Trust Centre for Human Genetics, University of Oxford, Oxford, United Kingdom; 2 Department of Statistics, University of Oxford, Oxford, United Kingdom; North Carolina State University, United States of America

## Abstract

Studies in model organisms suggest that epistasis may play an important role in the etiology of complex diseases and traits in humans. With the era of large-scale genome-wide association studies fast approaching, it is important to quantify whether it will be possible to detect interacting loci using realistic sample sizes in humans and to what extent undetected epistasis will adversely affect power to detect association when single-locus approaches are employed. We therefore investigated the power to detect association for an extensive range of two-locus quantitative trait models that incorporated varying degrees of epistasis. We compared the power to detect association using a single-locus model that ignored interaction effects, a full two-locus model that allowed for interactions, and, most important, two two-stage strategies whereby a subset of loci initially identified using single-locus tests were analyzed using the full two-locus model. Despite the penalty introduced by multiple testing, fitting the full two-locus model performed better than single-locus tests for many of the situations considered, particularly when compared with attempts to detect both individual loci. Using a two-stage strategy reduced the computational burden associated with performing an exhaustive two-locus search across the genome but was not as powerful as the exhaustive search when loci interacted. Two-stage approaches also increased the risk of missing interacting loci that contributed little effect at the margins. Based on our extensive simulations, our results suggest that an exhaustive search involving all pairwise combinations of markers across the genome might provide a useful complement to single-locus scans in identifying interacting loci that contribute to moderate proportions of the phenotypic variance.

## Synopsis

Although there is growing appreciation that attempting to map genetic interactions in humans may be a fruitful endeavor, there is no consensus as to the best strategy for their detection, particularly in the case of genome-wide association where the number of potential comparisons is enormous. In this article, the authors compare the performance of four different search strategies to detect loci which interact in genome-wide association—a single-locus search, an exhaustive two-locus search, and two, two-stage procedures in which a subset of loci initially identified with single-locus tests are analyzed using a full two-locus model. Their results show that when loci interact, an exhaustive two-locus search across the genome is superior to a two-stage strategy, and in many situations can identify loci which would not have been identified solely using a single-locus search. Their findings suggest that an exhaustive search involving all pairwise combinations of markers across the genome may provide a useful complement to single-locus scans in identifying interacting loci that contribute to moderate proportions of the phenotypic variance.

## Introduction

There is growing evidence supporting an important role for epistasis in the etiology of complex traits. Studies employing model organisms such as Drosophilla melanogaster and Saccharomyces cerevisiae (yeast) have suggested that epistasis occurs frequently, involves multiple loci, and in some cases produces effects as large as the main effects at the individual loci [1–4]. Although there is growing appreciation that searching for epistatic interactions in humans may be a fruitful endeavor, there is no consensus as to the best strategy for their detection, particularly in the case of genome-wide association (GWA) where the number of potential comparisons is enormous [5–7].

Recently, Marchini et al. [8] demonstrated that despite the considerable penalty introduced by multiple testing, it was possible to identify interacting loci that increased odds of disease using realistic sample sizes. Interestingly, searching over all possible pairs of loci was often more powerful than performing a single-locus scan across the genome in cases where the loci interacted epistatically. However, Marchini et al. examined three underlying disease models which represent only a small fraction of the entire parameter space of possible models. Additionally, Marchini et al. explicitly avoided models with little or no main effects at the margins. In these situations, single-locus searches are likely to fail and pairwise or higher-order searches will be necessary in order to detect loci [9–11]. A number of recent studies in humans and animals have identified loci that interact significantly but contribute little or no effect at the margins [12–29]. Such scenarios are challenging and worthy of further investigation since much of the current gene mapping methodology depends on the assumption of non-negligible main effects. If models that exhibit negligible marginal effects are common, then this will have significant consequences for how we go about searching for the genetic basis of complex phenotypes.

Even if testing all possible pairwise comparisons can be justified on theoretical grounds, there are still a number of practical difficulties associated with performing such a large number of statistical tests (i.e., storage requirements, computation time, etc.). For example, a smallish scan consisting of only 100,000 markers would entail ^100,000^C_2_ (i.e., 100,000 “choose” 2), or approximately 4.9 × 10^9^ comparisons, which might take several days on a typical workstation. Thus, rather than testing all possible pairwise comparisons, a more practical strategy might be to examine a subset of loci which could influence the trait. An obvious method of selecting loci is to evaluate their performance first on a single-locus test of association. That is, loci that meet some low threshold in a single-locus test are subsequently followed up in a two-locus analysis [8]. Intuitively, such an approach might also provide an advantage in power since the penalty due to multiple testing would not be as great as in an exhaustive pairwise search. We therefore investigated the performance of two simple two-stage strategies. In the first strategy, only loci that met an initial threshold were included in subsequent testing. In the second strategy, any locus that met the first-stage threshold was subsequently tested with all other markers across the genome regardless of whether these other markers met the initial threshold.

In summary, our manuscript expands on earlier work by (a) examining the performance of a two-locus and single-locus search across the genome for an extensive range of genetic models, (b) investigating the performance of each of these strategies using quantitative rather than disease traits, and (c) characterizing the performance of two, two-stage strategies which reduce the computational and multiple testing burden associated with an exhaustive two-locus search across the genome. Based on our extensive simulations, we demonstrate that an exhaustive two-locus search is more powerful than a single-locus strategy when loci interact for many of the situations considered, and is capable of detecting interacting loci that contribute to moderate proportions of the phenotypic variance using realistic sample sizes. In addition, we also show that an exhaustive search involving all pairwise combinations of markers across the genome is preferable to analyzing the data using a two-stage procedure that first conditions on one or both of the loci meeting some marginal level of significance in a single-locus test. Our results suggest that an exhaustive pairwise search of markers across the genome may provide a useful complement to single-locus scans in identifying interacting loci that contribute to moderate proportions of the phenotypic variance.

## Results

**Figure 1:**
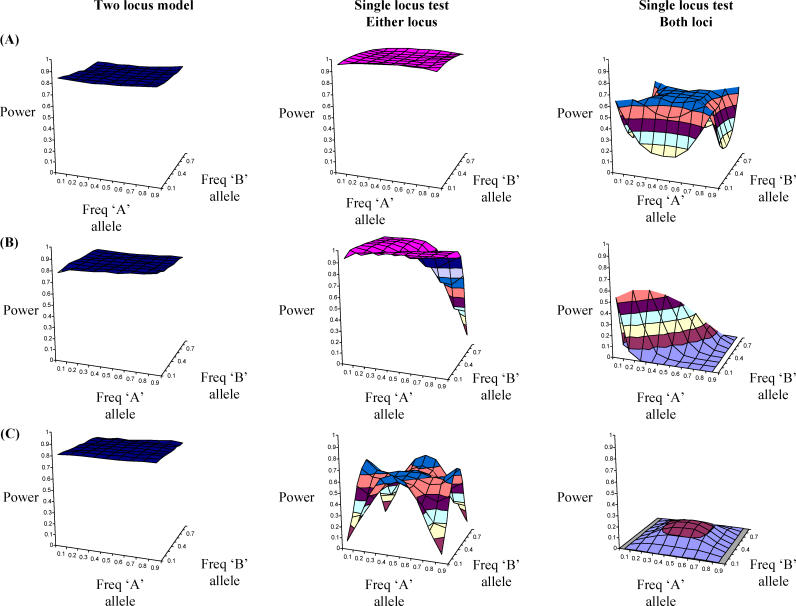
Power to Detect Association for 1,500 Individuals where Both Loci Are Responsible for 5% of the Trait Variance Power is displayed on the vertical axis, allele frequencies at both loci on the horizontal axes. Results are shown for an (A) additive model where there is no epistasis (MA), (B) a simple looking epistatic model (M27) in which an individual requires at least one copy of the increaser allele at both loci in order to increase the quantitative phenotype above baseline levels, and (C) a more exotic model where an individual has to be heterozygous at both loci in order to have a trait mean above baseline (M16).

Figure 1 presents an illustrative selection of results in order to highlight some general features of the data (a full list of results for all models and conditions can be found in Dataset S1). The first feature that should be apparent is that only 1,500 individuals are required in order to detect loci responsible for moderate proportions of the phenotypic variance (i.e., 5%) with appreciable power (approximately 80%) using an exhaustive pairwise search across the genome. As expected, when there was no interaction between the loci, the power to detect either locus using a single-locus search was greater than the power to detect both loci using the two-locus strategy. However, the power to detect both loci using a single-locus strategy was actually less than the power of the two-locus search—even when there was no epistasis, a result which also held with different numbers of markers across the genome (results not shown). Given this result, it is perhaps not surprising that when epistasis was present, the power to detect both loci was always greatest using the two-locus strategy. This reflects the usual situation in statistics where the most powerful test is the one that encompasses the true underlying model.

However, much more interesting is the comparison between the power of the two-locus search and the power to detect *either* locus via single-locus scan (Figure 1). For many of the models, the power to detect *either* locus using a single-locus strategy was greater than the power of a two-locus search for the majority of allele frequencies considered. However, for a small to moderate proportion of the space of possible allele frequencies, a two-locus strategy actually performed better than a single-locus search. These situations represent cases where the combination of allele frequencies is such that the majority of the genetic variance resides in the epistatic variance component, and hence the loci cannot be identified via single-locus tests of association (see below). Interestingly, Figure 1 also shows that for a small number of models, the power to detect *either* locus using a single-locus strategy is actually less than the power of a two-locus search for the majority of the parameter space of allele frequency combinations. These models tended to be the more exotic looking ones (e.g., M170, which requires an individual to be heterozygous at one locus and homozygous at the other in order to display the increased phenotype). Models such as these are difficult to explain via simple additive and dominance effects and thus are not amenable to single-locus tests of association.

**Figure 2:**
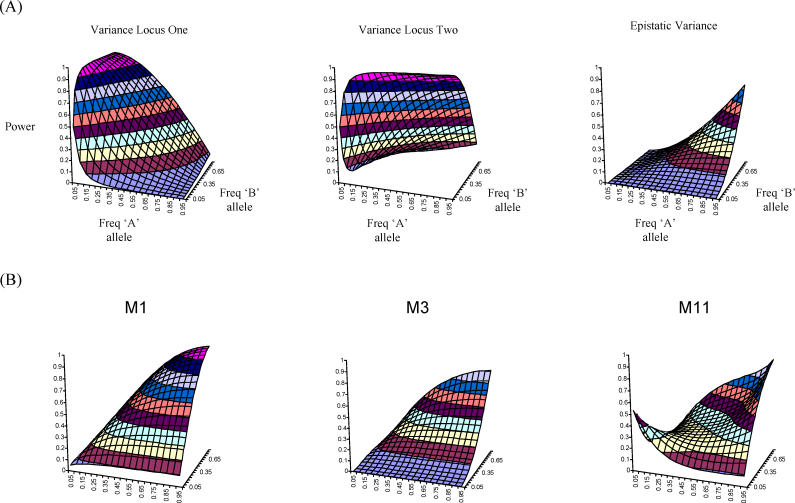
Partitioning of the Variance for Four Quantitative Trait Models (A) Expected variance components at locus 1, locus 2, and the epistatic variance component for model M27 in which an individual requires at least one copy of the increaser allele at both loci in order to increase the quantitative phenotype above baseline levels. Note in particular that when alleles “A” and “B” are common at both loci, the majority of genetic variance resides in the epistatic component. (B) The proportion of the total genetic variance which is due to statistical epistasis for three simple models. The vertical axis denotes the proportion of variance, whereas the horizontal axes denote allele frequencies at each locus. For each model, the epistatic variance component is appreciable for a sizeable portion of the space of possible allele frequencies.

Figure 2 illustrates a partitioning of the variance for a simple quantitative trait model (M27). Under this model, an individual requires at least one copy of the increaser allele at both loci in order to increase the quantitative phenotype above baseline levels (one could imagine how this could occur via simple biological process). Notice in particular how the proportion of the genetic variance in each of the different components varies with changes in the allele frequencies. For example, when the “A” and “B” alleles are both common, the majority of variance resides in the epistatic component, and there is little effect at the margins. In this situation, single-locus tests of association might fail to detect the loci even though they clearly influence the quantitative trait. It is only when a two-locus model which explicitly models the interaction between both contributing loci is fit to the data that the true underlying relationship becomes apparent and both loci can be identified [8]. In fact, it is sobering to realize that many simple-looking models (e.g., M1, M3, etc.) also contain sizeable regions of the space of possible allele frequencies where the epistatic variance component is appreciable (see the bottom of Figure 2 for some more examples). The implication is that these situations might also be common in reality and therefore frustrate attempts to localize genes in human populations via simple single-locus approaches.

**Figure 3:**
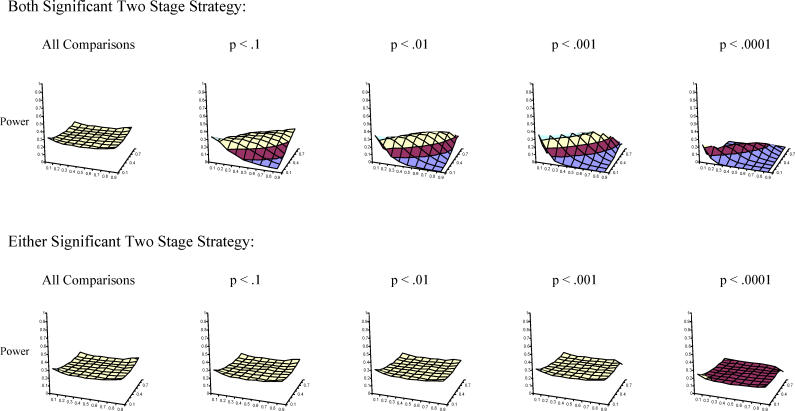
Power Associated with the Two-Stage Strategies in the Case of Two Loci Responsible for 5% of the Trait Variance Generated under Model M27 in which an Individual Requires at Least One Copy of the Increaser Allele at Both Loci in Order to Increase the Quantitative Phenotype above Baseline Levels as Applied to 1,000 Individuals Five different first-stage thresholds are shown. In the case of the Both Significant Two-Stage Strategy, there is a rapid decrease in power with increasingly stringent first-stage thresholds, except for a small proportion of the space of possible allele frequencies where there is a small gain in power. In the case of the Either Significant Strategy, power declines more slowly as the first-stage threshold becomes increasingly stringent.

Figure 3 illustrates the performance of each of the two-locus strategies for the same model depicted in Figure 2, which is also representative of many of the situations considered. In the case of the Both Significant Two-Stage Strategy (i.e., where both loci were required to meet a first-stage threshold in the single-locus scans in order to test the full interaction model), as the threshold became increasingly stringent, there was a decrease in power across the majority of the space of possible allele frequencies. However, for a small proportion of this space (i.e., when the allele frequencies at both loci were similar in the case of this model), there was a small increase in power, so long as the first-stage threshold was not too strict (the exact level varied across models). This pattern of results was similar across all of the models considered. In contrast, the power of the Either Significant Two-Stage Strategy (i.e., where only one locus was required to meet a first-stage threshold in order to test the full interaction model) was dependent on the type of model under which the data were simulated. For example, in the case of more exotic models (e.g., M170), there was a decrease in power across the majority of the space of possible allele frequencies even at liberal first-stage cutoff levels (i.e., *p* < 0.5). For other models, power remained relatively constant across the space of possible allele frequencies before decreasing at more stringent cutoffs (the exact threshold that this decline occurred depended on the particular model simulated).

## Discussion

Our results indicate that despite the considerable statistical penalty introduced by multiple testing, it is possible to detect interacting loci responsible for moderate proportions of the phenotypic variance (i.e., 5%) using realistic sample sizes (approximately 1,500 individuals). This result is important because it was widely assumed prior to this and our previous study [8] that testing for pairwise interactions in GWA was a waste of time, simply because the large number of comparisons would necessitate a severe statistical correction. In contrast, we have provided several examples, where not only is it possible to detect pairwise interacting loci in GWA analysis, but where performing an exhaustive pairwise search of the genome can actually increase power to detect interacting loci which might not be identified using single-locus searches.

A particularly novel aspect of our study concerns the evaluation of two simple two-stage procedures which could be used to decrease the multiple testing burden associated with an exhaustive two-locus scan of the genome. Our simulations surprisingly demonstrate that each of these two-stage strategies was associated with a decrease in power to detect both loci relative to an exhaustive two-stage search. Why was this the case? The key to understanding these results lies in the expected variance components underlying the model under which the data were simulated. In order for the Both Significant Two-Stage Strategy to succeed, *both* loci must pass the first-stage threshold in the single-locus test of association. For this to occur, some of the genetic variance must be present in *both* single-locus components. Using the example in Figures 2 and 3 as an illustration, this was not the case for the majority of the space of possible allele frequencies, and therefore the strategy tended to perform poorly (as was the case for all models except for the purely additive model MA). It was only when the frequency of the increaser alleles at both loci was low, that the genetic variance was divided equally between the two single-locus components. In this situation, both loci were correctly identified at stage 1 and there was a small increase in power due to the fewer tests performed.

In order for the Either Significant Two-Stage Strategy to succeed, some of the genetic variance must be present in *at least one* of the single-locus components. This is because a locus identified using this strategy is subsequently tested with all other markers across the genome regardless of whether they meet the first-stage threshold. In other words, the Either Significant Two-Stage Strategy will fail when the majority of the genetic variance is located in the epistatic variance component or when the first-stage threshold is so stringent that both of the single-locus components cannot be resolved with adequate power. Such situations occurred particularly under extreme scenarios (e.g., M170) but also for proportions of the space of possible allele frequencies in other simpler models (see, e.g., Figure 2 for examples). It is also important to note that although the Either Significant Two-Stage Strategy led to some gains in computation time, storage, etc., overall the method did not increase the statistical power to detect interacting loci. This was because the need to condition on one of the loci being significant in the first stage of testing offset any gains due to the decreased number of tests performed.

Our results imply that, practical considerations aside, it is preferable to perform an exhaustive two-locus search across the genome rather than either of the two-stage procedures that we examined. Otherwise, investigators risk discarding significant loci that only exhibit small effects at the margins. Although it is unclear to what extent multilocus interactions contribute to complex traits in humans, a number of recent studies employing computationally intensive methods have found evidence of statistical epistasis in the absence of significant marginal effects [27–29]. It has even been suggested that undetected epistasis may be one reason why single-locus tests of association fail to replicate across sample groups [8,30]. Since in the presence of epistasis any polymorphism must be considered in the context of other polymorphisms, if only single-locus tests are conducted, then a significant association may only be apparent when the allele frequencies in that sample are conducive to seeing a main effect [30].

Carlborg and colleagues reached similar conclusions but in the context of genome-wide linkage analysis of experimental line crosses [21,31–35]. Results from these designs are particularly relevant to our study since single-locus and epistatic effects can be modeled orthogonally [36–38] as they are in GWA. For example, Carlborg and Haley [32] advocate a two-locus search of the genome in preference to a forward selection procedure which conditions on significant marginal effects at the individual loci, the rationale being that loci which only contribute to epistatic effects might otherwise be missed [21,31–35]. Consistent with this view, a growing number of linkage studies in experimental animals have identified loci that interact significantly but contribute little or no effect at the margins [12–26].

We note that this situation is different from genome-wide linkage analysis in human (and other randomly mating) populations where single-locus and epistatic effects are partially confounded [39]. This is essentially because the allele sharing variables (identity by descent coefficients) that index epistatic and nonepistatic effects are correlated. As a consequence, a single-locus linkage model will absorb some of the variance due to epistatic effects, even though the power to detect epistasis formally through a multilocus linkage model might be low. Thus, a single-locus test of linkage might still be able to detect a locus that has little or no main effect but interacts epistatically with another (possibly) unmeasured locus [39]. In this vein, a sequential search for interacting loci seems like an ideal strategy in these situations. However, as we have shown in our simulations, a sequential search will not necessarily work in GWA as interacting loci that do not display large main effects may be missed.

Finally, we note that several groups are in the process of developing sophisticated algorithms which hold promise for the detection of even higher-order epistatic interactions [27,29–31,34,35,40]. For example, Millstein et al. [40] recently developed a strategy for detecting interacting loci in case control studies which they call a “focused interaction testing framework.” Their basic idea is to perform likelihood ratio tests in stages that increase in complexity with the order of the interaction considered. Joint tests of main effects and interactions are performed conditional on significant lower-order effects. The authors achieve a reduction in the total number of tests performed by first screening potential gene combinations with a goodness of fit chi-square statistic that depends on association among candidate genes in the pooled case control group. Through simulation they demonstrate that their focused interaction testing framework approach has good power to detect interacting loci and is more powerful than performing marginal tests of candidate genes under a range of situations [40]. We note that while their strategy holds great promise for the analysis of interacting candidate genes, the challenge remains to develop algorithms that will efficiently detect higher-order epistatic interactions in GWA analysis where the number of comparisons is much greater.

In conclusion, we have documented an extensive range of examples in which an exhaustive search involving all pairwise combinations of markers across the genome shows favorable performance for identifying interacting quantitative trait loci that contribute to moderate proportions of the phenotypic variance. Such a strategy can produce superior results to a simple single-locus search in situations where loci interact and is more powerful than a two-stage approach. With the era of GWA fast approaching, we believe that an exhaustive pairwise analysis may prove a useful complement to single-locus scans in shedding light on the genetic etiology of complex traits.

## Materials and Methods

### Simulations.

**Figure 4:**
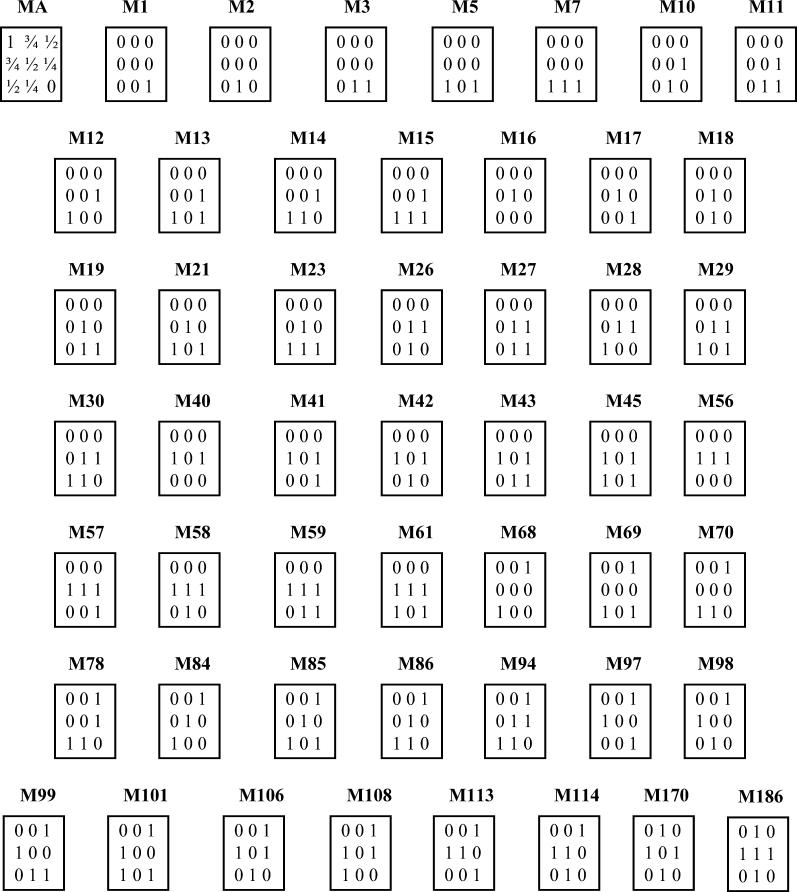
Fifty-One Biallelic Two-locus Models Incorporating Varying Degrees of Epistasis Each row indexes the genotype at the first locus and each column refers to the genotype at the second locus. The number in each cell is the trait mean for that genotype. Models range from the very simple (e.g., M1) to the more exotic (e.g., M170).

We considered an extensive range of quantitative trait models that incorporated varying degrees of epistasis (Figure 4). Our focus was on models with a specific set of phenotypic means (i.e., 0 or 1) in order to allow comparison with binary disease models. Although there are 2^9^ = 512 possible models, because of symmetries in the data, only 50 of these are unique [41]. These models include scenarios where it is easy to imagine a simple molecular basis for the interaction (e.g., M1), as well as more exotic situations which would require a more complicated biological explanation (e.g., M170). We also simulated under a purely additive model (“MA” in Figure 4) in order to contrast the case where there was no interaction between the loci. Data were simulated according to each model over a range of allele frequencies at both loci (i.e., 0.1, 0.2, …, 0.9). Generating the data in this way allowed the size of the effect at the margins to vary and also the relative importance of epistatic effects while maintaining the total genetic variance at a constant. We simulated cases where the two loci were responsible for 5% of the trait variance, and simulated 1,000, 1,200, or 1,500 unrelated individuals. We assumed perfect LD between trait and marker loci and, in order to correct for multiple testing, assumed the existence of an additional 100,000 markers across the genome (i.e., even though only two loci were in fact simulated in reality).

### QTL detection.

Initially we were most interested in whether it would be possible to detect interacting loci using realistic sample sizes, and whether there might be situations where undetected epistasis adversely affected power to detect association when only single-locus approaches were used. We therefore calculated the power to detect association using two different strategies: a locus-by-locus search using single-locus tests of association and an exhaustive two-locus search fitting two-locus models of association to all pairwise combinations of markers. For power comparisons, because the two-locus strategy considers two QTLs simultaneously, it is directly comparable to a single-locus approach that defines success on the basis of detecting either locus individually. We also compared the two-locus strategy with a more stringent single-locus search in which both of the interacting loci needed to be detected in order for the test to be declared significant.

Tests of association were fit to the data via maximum-likelihood assuming an underlying multivariate normal probability density function (we note that similar results were obtained using least squares regression). The log-likelihood *(l)* for individual *i* was calculated as:

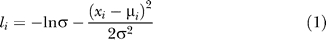
where x*_i_* is the trait value of the *i*th individual, *μ_i_* is the maximum likelihood estimate of the mean of individual *i* according to the underlying genetic model being fit, and *σ* is the maximum likelihood estimate of the residual standard deviation not accounted for by the model. For example, in the case of the full two-locus model, nine different *μ_i_* values were estimated corresponding to each of the nine possible two-locus genotypic means. In the case of the single-locus models, three different *μ_i_* values were estimated corresponding to each of the three marginal genotypic means, and, similarly, in the case of the null model of no association, *μ_i_* was the overall mean of the trait values in the sample regardless of the genotypes at the two loci. We note that in all instances the maximum likelihood estimate of each parameter is simply its sample value (i.e., the sample genotypic mean or variance) which can be calculated quickly and efficiently in closed form without the need for optimization.

Significance was assessed by computing the difference in −2 log-likelihood χ^2^ between models, with the degrees of freedom for these tests equal to the difference in df between the full and submodels. For example, since ten parameters are estimated under the full two-locus model (i.e., nine genotypic means plus a single residual variance) and only two parameters are estimated under the null model (i.e., an overall mean and a residual variance), the full two-locus test involves eight degrees of freedom. Similarly, since four parameters are estimated under the single-locus model, the single-locus test of association involves two degrees of freedom. These numbers of course assume that all the relevant genotypic combinations are present in the data. In some circumstances, one or more genotypic combinations were not present in the simulated data in which case the degrees of freedom of the test were adjusted accordingly. In all cases, simple Bonferroni corrections were used to account for multiple testing (0.05/L in the case of the single-locus strategy and α/^L^C_2_ in the case of the two-locus search where L = 100,000 is the number of markers). Power was computed via 10,000 replicates.

We also determined the power of two simple two-stage strategies. We refer to the first of these strategies as the “Both Significant Two-Stage Strategy” and the second as the “Either Significant Two-Stage Strategy.” In the first stage of both procedures, we identified all loci that were significant in single-locus tests (as above) at one of seven liberal thresholds (i.e., α_1_ = 0.5, 0.1, 0.05, 0.01, 0.001, 0.0005, 0.0001). We called this set of loci *I*_1_ ⊆ {1,…*L*}. We let *d_1_* be the degrees of freedom of the single-locus model fitted at stage 1 for locus *l* (maximum 2 degrees of freedom if all three genotypes are present) and defined *k_l_* such that
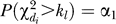
 for *l* ⊆ *I*_1_. In the case of the Both Significant Two-Stage Strategy, for each pair of loci *l* and *m* identified in stage 1 (*l, m ∈ I*_1_, *l* ≠ *m*), we calculated the log-likelihood ratio statistic *R_(l,m)_* for the full interaction model. For the Either Significant Two-Stage Strategy, we calculated the log-likelihood ratio statistic *R_(l,m)_* associated with the full interaction model for each locus identified in stage 1 with all other loci (regardless of whether these loci met the first-stage significance threshold). Because of the way in which loci *l* and *m* were identified, *R_(l,m)_* > *k_l_* + *k_m_* (where *k_m_* = 0 in the case of the Either Significant Two-Stage Strategy). We therefore defined a new statistic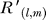 = *R_(l,m)_* – (*k_l_* + *k_m_*) and assessed the significance of this statistic against a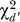 distribution in which *d*′ is the degrees of freedom of the full model fitted at the two loci. In the tests, the null hypothesis being tested is that both loci are not associated with the phenotype. We set the level of significance using a Bonferroni correction based on the expected number of tests to be performed, yielding the same overall error rate in each strategy, (α/^*α*_1_^ ^*L*^ *C*_2_) in the case of the Both Significant Two-Stage Strategy and [α/(*^L^C*_2_ − ^(*L − α*_1_^*L*)^^ *C*_2_)] in the case of the Either Significant Two-Stage Strategy. Through simulation we found this procedure to provide an accurate test of interaction between two loci [8].

### Partitioning the model into expected variance components.

In order to better understand the performance of the different strategies, it is instructive to consider how the total genetic variance for any two-locus model can be divided into three mutually exclusive components: the genetic variance at locus 1, the genetic variance at locus 2 and the epistatic (or interaction) variance (the derivation of these components is well known [42–46] and we refer the interested reader to these articles as well as several excellent texts which cover the subject [47,48]). The partitioning is important because it represents the amount of genetic variance (and hence statistical power) which can be captured by a single-locus test of association at locus 1, a single-locus test of association at locus 2, and the amount of genetic variance which cannot be captured by single-locus tests, respectively. The single-locus components represent the effects of a locus averaged over all other loci (i.e., the ‘marginal' effects), and are equivalent to the combined additive and dominance components of classical quantitative genetics [49]. The epistatic component arises because of nonadditive interactions between the loci and represents the remainder of the genetic variance which is not accounted for by the single-locus components. The sum of all three components is the amount of genetic variance which can be captured by a two-locus test of association.

To illustrate this partitioning formally, consider the 3 × 3 matrix of genotypic means and their frequencies in Table 1. Each row displays the genotype at the first locus, whereas each column indexes the genotype at the second. Each cell contains a genotypic mean and its respective frequency (under Hardy-Weinberg equilibrium). Given the genotypic means and frequencies at both loci, it is possible to calculate the mean (μ) and total genetic variance of the system (σ^2^). The mean is the sum of all genotypic means weighted by their frequencies:

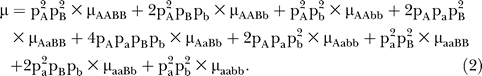
The total genetic variance is equal to each genotypic mean minus the overall mean (μ), squared, and then summed over all possible genotypes weighted by the appropriate genotypic frequency:

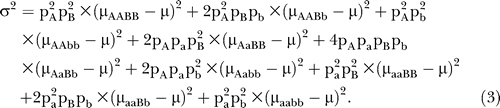
The amount of genetic variance at locus 1 (or locus 2) may be calculated by subtracting the mean for each marginal genotype from the overall mean, squaring and then summing over all possible genotypes weighted by the relevant genotypic frequency:

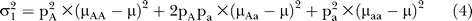


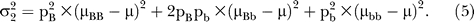
The epistatic variance is simply the amount of genetic variance not accounted for by the single-locus components:

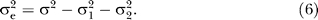
We performed this partitioning for all 51 models in Figure 4 in order to gain a better insight into the relative performance of each of the different GWA strategies.

**Table 1:**
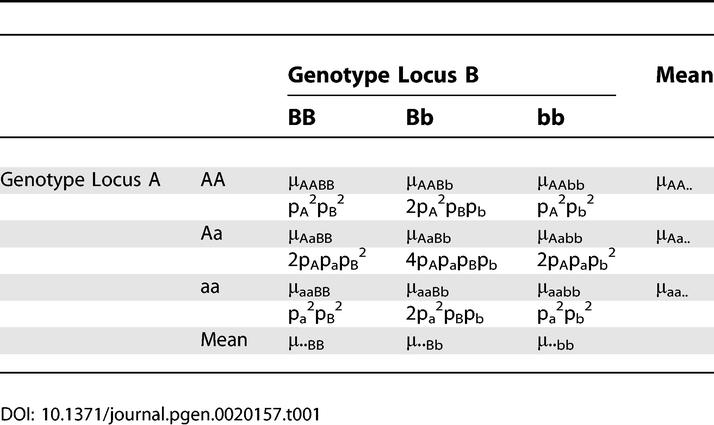
Genotypic Means and Frequencies for a Biallelic Two-Locus Model

## Supporting Information

Dataset S1 Supplementary Results (4.9 MB XLS)

## Abbreviation

GWA: genome-wide association
